# Ignorance can be evolutionarily beneficial

**DOI:** 10.1002/ece3.3627

**Published:** 2017-11-23

**Authors:** Jared M. Field, Michael B. Bonsall

**Affiliations:** ^1^ Wolfson Centre for Mathematical Biology Mathematical Institute University of Oxford Oxford UK; ^2^ Mathematical Ecology Research Group Department of Zoology University of Oxford Oxford UK

**Keywords:** Bayes’ theorem, ignorance, information, sleep, statistical decision theory

## Abstract

Information is increasingly being viewed as a resource used by organisms to increase their fitness. Indeed, it has been formally shown that there is a sensible way to assign a reproductive value to information and it is non‐negative. However, all of this work assumed that information collection is cost‐free. Here, we account for such a cost and provide conditions for when the reproductive value of information will be negative. In these instances, counterintuitively, it is in the interest of the organism to remain ignorant. We link our results to empirical studies where Bayesian behavior appears to break down in complex environments and provide an alternative explanation of lowered arousal thresholds in the evolution of sleep.

## 1 INTRODUCTION

In all areas of biology, observations or cues can elicit a change in behavior or phenotype. For example, the duration of daylight hours affects the flowering time of plants (Amasino, 2010), chemotactic gradients provide a way for bacteria to locate favorable environments (Adler, 1966), and the sighting of a predator may cause an animal to flee. In each case, the observation aids in the choice of an action that will benefit the organism. In this way, if there is no cost in collecting information, then organisms should always collect it. Indeed, borrowing from economic theory, but replacing utilities with reproductive values, it has been shown that the reproductive value of information is always non‐negative (McNamara & Dall, 2010; Pike, McNamara, & Houston, 2016). This remarkable result suggests that organisms can never decrease their fitness by being more informed. This has far‐reaching implications in areas of biology as diverse as public goods games, foraging, collective behavior, and sleep. However, in reality, information comes at a cost (Laughlin, de Ruyter van Steveninck, & Anderson 1988). In this study, we investigate the consequences of formally including such costs in the current theoretical framework. While information may be inherently valuable in decision‐making, we challenge the view that it always should be or indeed is collected. Indeed, we suggest that to understand behavior more fully any valuations of information should not be separated from the associated costs of collection. Natural selection acts on the process as a whole and so should be considered as such.

In the context of organismal biology, information use has been approached from two very distinct angles. The first employs the information theory pioneered by Shannon and Weaver (1949). The alternative, which we take, makes use of statistical decision theory (Dall, Giraldeau, Olsson, McNamara, & Stephens, 2005; McNamara, Green, & Olsson, 2006; McNamara & Houston, 1980). The former focuses on uncertainty reduction, whereas the latter considers how information updating, via Bayes rule, explicitly affects fitnesses. Only recently was the connection between these two approaches shown (Donaldson‐Matasci, Bergstrom, & Lachmann, 2010). Strikingly, mutual information (an information‐theoretic measure which quantifies the uncertainty of an outcome after an observation) provides an upper bound on the value of information (a decision‐theoretic measure expressed explicitly in terms of fitnesses) (Donaldson‐Matasci et al., 2010). However, this is only the case when the fitness measure used is long‐term lineage growth rate. Here, we use individual reproductive values. In this way, the value of information is defined by taking the difference in expected optimal reproductive values before and after collecting information. The literature on information use in biology is vast so we do not try to cover it here. However, for useful reviews, see (Dall et al., 2005; Schmidt, Dall, & Van Gils, 2010; Seppänen, Forsman, Mönkkönen, & Thomson, 2007; Valone, 2007).

We start by adopting the same framework as in McNamara & Dall (2010). Following this, we derive conditions, in the absence of a cost, for when the reproductive value of information is precisely equal to zero. In this case, an organism that collects information will be no better off than one that does not. Next, we account for a cost of collecting information. We do this by discounting the previous reproductive values associated with certain actions and beliefs prior to information collection. This way, the cost is due solely to the collection of information itself. In the proceeding section, we show that the same conditions that we derived previously now lead to the reproductive value of information being negative. In this instance, an organism that remains ignorant will have a higher fitness than one that does not. This has implications for many empirical studies whereby, in increasingly complex environments, organisms were found to stop behaving in a Bayesian manner (Valone, 2006). It may be that the cost of collection in these complex environments outweighs any benefits. Additionally, we consider the ramifications for evolutionary problems such as the evolution of sleep. In particular, we suggest that the lowered arousal thresholds associated with sleep, often explained as the by‐product of other vital functions, may instead be accounted for by our results (Lima & Rattenborg, 2007). We finish by summarizing our findings and suggesting future directions of work relating ignorance at the level of the individual to behavior at the level of the group (Couzin et al., 2011).

## 2 FRAMEWORK

Following (McNamara & Dall, 2010), we start by assuming there are *n* possible true states of nature θ_n_, collected in the vector Θ = (θ_1_, …, θ_*n*_), about which a given organism is unsure. This organism believes, with probability *p*_*i*_, that θ_*i*_ is the true state. In this way, the vector *P *= (*p*_1_, …, *p*_*n*_) summarizes the organism's (imperfect) knowledge of nature.

Further, we assume that this organism must take a certain action, given its beliefs, which will affect its fitness. In particular, a certain action *u* will have reproductive value *V*_*i*_(*u*) given that the true state of nature is θ_*i*_. With this setup, we find the expected reproductive value of action *u* to be given by

(1) $$V\left(u,P\right)=\sum_{i=1}^{n}p_{i}V_{i}\left(u\right).$$

Then for any vector *P*, we define the optimal action u* so that

(2) $$V\left(u,P\right)\leq V\left(u^{*},P\right),$$

for any action *u*.

We now suppose that this organism can gather information, thereby updating its knowledge. We assume it does this by sampling a random variable *X*, which depends on Θ. We denote the probability the observed value of *X* is *x* given θ_*i*_ by *f*(*x*|θ_*i*_). With this, we can interpret *p*_*i*_ to be a prior probability and use the observation to form its posterior using Bayes rule, which we denote by *q*_*i*_(*x*). Doing so gives

(3) $$q_{i}\left(x\right)=\frac{p_{i}f\left(x|\theta_{i}\right)}{\sum_{j=1}^{n}p_{j}f\left(x|\theta_{j}\right)}.$$

With this extra information, the organism's knowledge is now summarized by *Q*(*X*) = (*q*_1_, …, *q*_*n*_).

With this setup, we define the expected reproductive value (taken over observations *X*) of information *I* such that (McNamara & Dall, 2010):

(4) $$I=E\left[V\left(\tilde{u}\left(X\right),Q\left(X\right)\right)\right]-V\left(u^{*},P\right).$$

The first term of this quantity is the reproductive value after collecting information, optimized over actions, and averaged over observations. The second term is the optimal reproductive value if information is not collected. In this way, the difference quantifies the benefit of collecting information.

## 3 EXPECTED VALUE OF INFORMATION IS NON‐NEGATIVE

While inequality (2) is expressed in terms of *P*, it is also true for the random vector *Q*(*X*). In particular,

(5) $$V\left(u,Q\left(X\right)\right)\leq V(\tilde{u}\left(Q\left(X\right)\right),Q\left(X\right)).$$

Taking the expected value with respect to *X*, we then have that

(6) $$E\left[V\left(u,Q\left(X\right)\right)\right]\leq E[V\left(\tilde{u}\left(Q\left(X\right)\right),Q\left(X\right)\right)].$$

The left‐hand side of inequality (6) can be rewritten as

(7) $$\begin{array}{c} E\left[V\left(u,Q\left(X\right)\right)\right]\;=\;E\left[\sum_{i=1}^{n}q_{i}\left(X\right)V_{i}\left(u\right)\right], \end{array}$$

(8) $$\begin{array}{c} =\;\sum_{i=1}^{n}E\left[q_{i}\left(X\right)V_{i}\left(u\right)\right], \end{array}$$

(9) $$\begin{array}{c} =\;\sum_{i=1}^{n}E\left[q_{i}\left(X\right)\right]V_{i}\left(u\right). \end{array}$$

However, as the expectation is taken over observations *x*, we have

(10) $$E\left[q_{i}\left(X\right)\right]\;=\;\sum_{x}q_{i}\left(x\right)f\left(x\right),$$

where *f*(*x*) is the probability that the observed value of *X* is *x*. Using the law of total probability, *f*(*x*) can in fact be written as

(11) $$f\left(x\right)=\sum_{j=1}^{n}p_{j}f\left(x|\theta_{j}\right),$$

so that, coupled with (3), (10) can be reexpressed as

(12) $$\begin{array}{c} E\left[q_{i}\left(X\right)\right]\;=\;p_{i}\sum_{x}f\left(x|\theta_{j}\right), \end{array}$$

(13) $$=\;p_{i},$$

as *f*(*x*|θ_*j*_) is a probability distribution.

Now, using (13) in (9), we find that

(14) $$\begin{array}{c} E\left[V\left(u,Q\left(X\right)\right)\right]\;=\;\sum_{i=1}^{n}p_{i}V_{i}\left(u\right), \end{array}$$

(15) =V(u,P),

by (1). This statement is true for any action *u* so that it is in particular true of the action u*(*P*) that optimizes *V*(*u*,* P*).

Finally, using (15) in inequality (6) and rearranging we find that

(16) $$0\;\leq \;E\left[V\left(\tilde{u}\left(Q\left(X\right)\right),\;Q\left(X\right)\right)\right]\;-\;V\left(u^{*},P\right).$$

Otherwise put the expected reproductive value of information *I* is non‐negative.

## 4 WHEN IS THE EXPECTED VALUE OF INFORMATION EQUAL TO ZERO?

So far, we have shown (and also in (McNamara & Dall, 2010)) that the expected value of information is non‐negative. However, if it is equal to zero, then an organism that collects information will be no better off than an organism that does.

To investigate this, we start by bounding inequality (6) from above. Similar to (7) and (8) but for the right‐hand side of (6), we have

(17) $$\begin{array}{c} E\left[V\left(\tilde{u}\left(Q\left(X\right)\right),Q\left(X\right)\right)\right]\;\;=\;\;E\left[\sum_{i=1}^{n}q_{i}\left(X\right)V_{i}(\tilde{u}\left(X\right)\right], \end{array}$$

(18) $$=\sum_{i=1}^{n}E\left[q_{i}\left(X\right)V_{i}\left(\tilde{u}\left(X\right)\right)\right].$$

However, this time as the action *u*(*X*) taken depends on the observation we must take the expectation directly. In particular, we have

(19) $$E\left[V\left(\tilde{u}\left(Q\left(X\right)\right),Q\left(X\right)\right)\right]=\sum_{i=1}^{n}\sum_{x}q_{i}\left(x\right)V_{i}\left(\tilde{u}\left(x\right)\right)f\left(x\right).$$

We now suppose that there exists some $\hat{x}$ such that

(20) $$\begin{array}{c} \sum_{i=1}^{n}\sum_{x}q_{i}\left(x\right)V_{i}\left(\tilde{u}\left(x\right)\right)f\left(x\right)\leq \sum_{i=1}^{n}\sum_{x}q_{i}\left(x\right)V_{i}\left(\tilde{u}\left(\hat{x}\right)\right)f\left(x\right) \end{array}.$$

Using (3), this then becomes

(21) $$\begin{array}{c} \sum_{i=1}^{n}\sum_{x}q_{i}\left(x\right)V_{i}\left(\tilde{u}\left(x\right)\right)f\left(x\right)\;\leq \;\sum_{i=1}^{n}p_{i}V_{i}\left(\tilde{u}\left(\hat{x}\right)\right)\sum_{x}f\left(x|\theta_{i}\right), \end{array}$$

(22) $$=V(\tilde{u}\left(\hat{x}\right),P),$$

as *f*(*x*|θ_*i*_) is a probability distribution. Hence, if such an $\hat{x}$ does exist, we have showed that

(23) $$V\left(u^{*},P\right)\leq E\left[V\left(\tilde{u}\left(Q\left(X\right)\right),Q\left(X\right)\right)\right]\leq V\left(\tilde{u}\left(\hat{x}\right),P\right).$$

However, from the very definition of u* it must be that

(24) $$\begin{array}{c} V\left(\tilde{u}\left(\hat{x}\right),P\right)\leq V\left(u^{*},P\right), \end{array}$$

so that in fact

(25) $$\begin{array}{c} E\left[V\left(\tilde{u}\left(Q\left(X\right)\right),Q\left(X\right)\right)\right]=V\left(u^{*},P\right), \end{array}$$

and so

(26) I=0.

For such an $\hat{x}$ to exist, we need, from (20), that

(27) $$\sum_{x}q_{i}\left(x\right)V_{i}\left(\tilde{u}\left(x\right)\right)f\left(x\right)\leq \sum_{x}q_{i}\left(x\right)V_{i}\left(\tilde{u}\left(\hat{x}\right)\right)f\left(x\right),$$

for each *i*. Using (3), this is equivalent to requiring

(28) $$\begin{array}{c} \sum_{x}f\left(x|\theta_{i}\right)V_{i}\left(\tilde{u}\left(x\right)\right)\;\leq \;V_{i}\left(\tilde{u}\left(\hat{x}\right)\right)\sum_{x}f\left(x|\theta_{i}\right), \end{array}$$

(29) $$=\;V_{i}\left(\tilde{u}\left(\hat{x}\right)\right),$$

which is in turn equivalent to

(30) $$f\left(\hat{x}|\theta_{i}\right)V_{i}\left(\tilde{u}\left(\hat{x}\right)\right)+\sum_{x\neq \hat{x}}f\left(x|\theta_{i}\right)V_{i}\left(\tilde{u}\left(x\right)\right)\leq V_{i}\left(\tilde{u}\left(\hat{x}\right)\right).$$

This statement will clearly be true in three cases. Either

(31) $$V_{i}\left(\tilde{u}\left(x\right)\right)=0,$$

for each $x\neq \hat{x}$ and for each *i* or

(32) $$f(x|\theta_{i})=0,$$

for each $x\neq \hat{x}$ and for each *i* or, finally, a mixture of these two previous extreme cases. In particular, we can have instances where there is a particular $x\neq \hat{x}$ so that

(33) $$V_{i}\left(\tilde{u}\left(x\right)\right)\neq 0,\;f\left(x|\theta_{i}\right)=0,$$

for each *i* or *vice versa* so that

(34) $$V_{i}\left(\tilde{u}\left(x\right)\right)\;=\;0,\;f\left(x|\theta_{i}\right)\neq 0,$$

for each *i*.

Condition (31) asserts that all other possible actions will have a reproductive value of zero. Though unlikely, this may be realized if presented with an extreme situation (such as certain death in the face of predation) when only one action will lead to survival.

Alternatively, via (11) condition (32) implies

(35) $$f(\hat{x})=1.$$

The interpretation here is that only the observation $\hat{x}$ will be observed. In this way, this condition says something about the environment in which an organism finds itself. If the organism finds itself in a period of relative constancy (such as certain safety due to a temporary lack of predators), this condition will be fulfilled.

The blended conditions (33) and (34) represent the more realistic situations where there is still flexibility in actions with nonzero reproductive value but the observation to which it corresponds will not be observed or where an observation will have nonzero probability of being observed, but the associated reproductive value is zero, respectively. Otherwise put, an organism may be adapted and able to respond to a certain cue; however, currently is in a situation where that cue will not be observed. Alternatively, the organism may observe a certain cue but is unable to respond adequately.

## 5 ACCOUNTING FOR A COST OF INFORMATION COLLECTION

We now assume that the collection of information comes at a cost *c*. The reproductive value associated with any given action *u* taken after gathering information will then be discounted by this cost. Hence, if an organism collects information, the reproductive value of action *u* given state θ_*i*_ will be

(36) $${\overset{¯}{V}}_{i}\left(u\right)=V_{i}\left(u\right)-c.$$

As before, we have that

(37) $$E\left[\overset{¯}{V}\left(u,Q\left(X\right)\right)\right]\;\leq \;E[\overset{¯}{V}\left(\tilde{u}\left(Q\left(X\right)\right),Q\left(X\right)\right)],$$

for any *u*. Replacing *V*_*i*_(*u*) with ${\overset{¯}{V}}_{i}\left(u\right)$ in (9), the left‐hand side of this inequality can be written as

(38) $$E[\overset{¯}{V}(u,Q(X))]=V(u,P)-c,$$

which is, in particular, true for u* so that we have

(39) $$V\left(u^{*},P\right)-c\leq E\left[\overset{¯}{V}\left(\tilde{u}\left(Q\left(X\right)\right),Q\left(X\right)\right)\right].$$

Similarly, replacing occurrences of *V*_*i*_(·) with ${\overset{¯}{V}}_{i}$ (·) in the entire preceding section, it follows that

(40) $$E\left[\overset{¯}{V}\left(\tilde{u}\left(Q\left(X\right)\right),Q\left(X\right)\right)\right]\;\;\leq \;\;V\left(u^{*},P\right)-c,$$

so that, if there is a cost to collecting information, we find that

(41) $$E\left[\overset{¯}{V}\left(\tilde{u}\left(Q\left(X\right)\right),Q\left(X\right)\right)\right]=V\left(u^{*},P\right)-c.$$

Hence

(42) I=−c.

This result, as before, relies on one of the four conditions (31), (32), (33), (34) being true. In this case, an organism that collects information will be worse off than an organism that does not.

## 6 ALTERNATIVE MEASURES FOR VALUING INFORMATION

With some important recent exceptions, it is not generally believed that organisms are behaving in a strictly Bayesian manner (Biernaskie, Walker, & Gegear, 2009; Louapre, Van Baaren, Pierre, & Van Alphen, 2011; Valone, 2006). Instead, it is suspected that they follow Rules of Thumb that approximate optimal Bayesian strategies (McNamara & Houston, 1980; McNamara et al., 2006). These rules are based on either the experience of an organism's ancestors (and so is genetically encoded), the experience of the organism itself or a combination of both (Giraldeau, 1997; McNamara et al., 2006; Valone, 2006). When these rules are based on the experience of the organism, they will be informed by typical observations. In some circumstances, the expected value (such as with the analysis performed above) will be a good indication of typical sampled values.

However, in other cases the expected value is in fact a very poor measure of typical values. To be more concrete, consider *G*(*X*) defined such that

(43) $$G\left(X\right)=V\left(\tilde{u}\left(Q\left(X\right)\right),Q\left(X\right)\right)-V\left(u^{*},P\right).$$

with probability density function *g*(*x*). Note that this random variable has the property that **E** [*G*(*X*)] = *I*.

If *g*(*x*) is a unimodal symmetric distribution, then *I* will be a decent indication of typical values. However, if *g*(*x*) is, say, multimodal or skewed, then *I* will be a poor measure. Moreover, if *g*(*x*) is positively skewed, then typical values of *G* may be negative. In this case, *even without a cost associated with the act of information collection*, it will not be beneficial for an organism operating under a rule of thumb to collect information.

Whether or not *g*(*x*) is skewed will depend on *f*(*x*), the distribution describing *X*. However, the random variable *X* describes the environment the organism is in. For this reason, it is highly plausible that *X* and so *g*(*x*) will change as a function of time. For example, it may be that predators are more prevalent at a certain time over the course of one day. In this case, the probability of observing a predator will also change over that period. So too, then, will *g*(*x*). In short, though we have adopted existing frameworks whereby the mean is used as a typical value, we point out that the mean is not always the best measure of centrality.

## 7 CASE STUDY: SLEEP

The behavioral definition of sleep (in contrast to the physiological definition) involves both lowered activity levels and lowered arousal thresholds (Cirelli & Tononi, 2008; Siegel, 2009). Without this second property, behavioral sleep cannot be distinguished from simple rest (Cirelli & Tononi, 2008). While the adaptive value of inactivity (e.g., minimization of movement when it is dangerous) has been widely appreciated for quite some time (Brown, 2000), a state of “vulnerable disconnect” proves more difficult to explain. In this way, sleep remains an evolutionary conundrum. We address this problem using the results we have just developed.

To this end, assume there are two states of nature θ_0_, when it is best to be inactive, and θ_1_, when the converse is true. Further, assume that an organism believes with probabilities *p* and 1–*p* that the true state is θ_0_ and θ_1_, respectively. Finally, let *u *=* *0 represent inactivity and *u *=* *1 the opposite. Our assumptions then read:

(44) $$V_{0}\left(0\right)>V_{0}\left(1\right),$$

and

(45) $$V_{1}\left(0\right)<V_{1}\left(1\right).$$

Using (1), we have that

(46) $$V\left(0,p\right)=pV_{0}\left(0\right)+\left(1-p\right)V_{1}\left(0\right),$$

(47) $$V\left(1,p\right)=pV_{0}\left(1\right)+\left(1-p\right)V_{1}\left(1\right).$$

An organism should be inactive if

(48) V(0,p)>V(1,p),

which, upon rearranging, is equivalent to

(49) $$p>\frac{V_{1}\left(1\right)-V_{1}\left(0\right)}{V_{0}\left(0\right)-V_{0}\left(1\right)+V_{1}\left(1\right)-V_{1}\left(0\right)}.$$

Hence, given a certain belief *p* it is clear when to be active or inactive. We reiterate, however, that this tells us nothing about when sleep is adaptive. To be able to discuss this, we additionally need to know when (if at all) it may be adaptive for an organism to disconnect from its environment. We model this by the act of collecting or not collecting information. From (42), it is clear that an organism will be better off if it stops collecting information if any of conditions (31), (32), (33), (34) are fulfilled. We focus attention on condition (33). If an organism finds itself in a period of relative constancy and therefore certainty (with respect to the pressures that determine whether it is best to be active or inactive, such as the presence of predators), then in addition to being inactive it will be beneficial to not collect information. In other words, sleep will be adaptive.

## 8 DISCUSSION

The notion of putting a value on information has existed in economic theory for quite some time (Gould, 1974). Despite this, analogous work in the context of animal behavior has taken a little longer to catch up. Recently, however, it was formally shown that the reproductive value of information is always non‐negative when there is no cost in its collection (McNamara & Dall, 2010). From this, it has been concluded that organisms should always collect information. This conclusion has since not been investigated much further in the literature. While costs associated with sampling have been considered in some evolutionary games (see (McNamara, Stephens, Dall, & Houston, 2009), where the costs are crucial to maintain a mix of strategies), the focus has not been on whether or not information should be collected in the first place.

Here, using the same framework, we derived particular conditions for when the value of information will be equal to zero. In such a case, an organism that collects information will in fact be no better off than one that does not. Following this, we explicitly accounted for a cost of collecting information. In reality, this may come in the form of energy, time or resources that could otherwise be spent on other vital biological functions (Laughlin et al., 1988). In this instance, we found that under the same conditions as before, the value of information (which should more realistically be discounted by costs) will now be negative. Thus, there will be times when the collection of information will have a negative impact on an organism's fitness.

These conditions can be broadly organized into three groups. First, if an organism finds itself (potentially temporarily) in a situation whereby only one action will lead to a nonzero reproductive value, then the value of information will be negative. Second, if it is such that only one observation will be observed (again, potentially temporarily), then an organism will not benefit from collecting information. Both of these cases, in the absence of a cost of collecting information, are emphasized informally in (Pike et al., 2016). The third, a mixture of the previous two conditions, is not. In this case, an organism can still be flexible in its actions, and there can still be variance in observations, and yet the value of information will be less than zero. To be more concrete, this third condition will be fulfilled if an organism may observe a certain cue but is unable to respond adequately or if it is adapted and able to respond to a certain cue however currently is in a situation where it will not be observed. In either case, an organism will do better if they remain relatively ignorant.

Though the results presented here are quite broad they, and in particular the last condition, have strong implications for the evolution of sleep and sleep‐like states. Broadly speaking, sleep can be defined physiologically (characterized by certain brain activities) or behaviorally (characterized by inactivity and arousal thresholds). Some organisms, like dolphins, sleep according to one definition but not the other (Siegel, 2009). It is, however, the behavioral definition, and in particular the lowered arousal thresholds, that presents the evolutionary puzzle. While the adaptive value of inactivity has been recognized for some time (lack of movement when it is dangerous or pointless to do so, etc.), this alone does not demonstrate any adaptive value of sleep. If it is only the inactivity aspect of sleep that is adaptive, why not simply rest? For this reason, lowered arousal thresholds also need to be considered. This vulnerable disconnect from an organism's environment, which distinguishes sleep from rest, is most often explained by assuming a priori that sleep serves some vital function for which this behavioral shutdown is necessary (Lima & Rattenborg, 2007). Here, however, we have shown that such an assumption is not necessary. If there is a cost to collecting information, then there will be times when it should not be collected. We are not, of course, suggesting that vital functions of sleep do not exist. However, our result opens up the possibility that the vital functions evolved after behavioral shutdowns and not the other way around.

In an empirical review of animal Bayesian updating, it was found that in simple environments all but one species performed consistently with Bayesian predictions (Valone, 2006). In complex environments, this was found to not be true. The explanation put forward was that in these complex environments it is more difficult, or takes longer, for the organism in question to successfully learn prior distributions. An alternative and complementary explanation, suggested by our results, is that the cost of collecting information may be too high in these environments. In other words, they are making a Bayesian decision to remain ignorant. It would be exceedingly interesting to test this hypothesis experimentally.

More generally, we have shown that periods of ignorance can lead to fitness benefits at the level of the individual. For future theoretical work, it would be fruitful to understand how this may translate to behavior at the level of the group. Recently, it has been shown how uninformed individuals can help democratic consensus be reached in the face of internal conflicts (Couzin et al., 2011). This particular study, however, makes no reference to individual fitnesses but is, in essence, mechanistic. It would be particularly interesting to see if, taking an evolutionary approach, similar conclusions can be found.

## CONFLICT OF INTEREST

None declared.

## AUTHORS’ CONTRIBUTIONS

JMF carried out the research. JMF and MBB wrote the manuscript.
